# Recent Legal Decisions

**Published:** 1913-04-19

**Authors:** 


### RECENT LEGAL DECISIONS.

The National Insurance Act, *inter alia*, provides that all persons of the age of sixteen and upwards who are employed within the meaning of the Act under any contract of service must be compulsorily insured. The question in the test case brought by the Scottish Insurance Commissioners against the Royal Infirmary of Edinburgh was whether the resident physicians and surgeons, clinical assistants, and supervisors of the administration of anæsthetics are employed by the Infirmary under a contract of service. The First Division of the Court of Session were of opinion that they are not. The relation between these doctors and the Infirmary is defined as amounting not to a contract of service, but to a contract for services, the dividing line being found in the matter of control. Had the business of the Infirmary managers been to treat patients, then they might be said to have control over the doctors, but the Court thought that all they have to do is to provide and maintain the hospital, the treatment of injuries and diseases being given by skilled persons either gratuitously or for a slight recognition. In these circumstances the relationship of employer and employee was found not to exist at all between the Infirmary and the doctors.

A not infrequent question in paternity cases is the length of the gestation period as bearing on the evidence of paternity. The matter has been exhaustively discussed by all the writers on medical jurisprudence, and considering what has been said by both judges and doctors the opinion may safely be ventured that neither in law nor in medical science is it possible to fix an actual number of days as the minimum or as the maximum period of the gestation. In our system the Court is left free to deal with each case in the light of the evidence submitted. These remarks occur to us in reading the decision in *Williamson* v. *McClelland*, 1913, 1 S.L.T. 289, in which it was proved that the last act of connection took place 306 days before the birth of the child, and yet the Court found that that was not in law an impossible period of gestation.

